# Comparison of sleep quality deterioration by subgroup of painful temporomandibular disorder based on diagnostic criteria for temporomandibular disorders

**DOI:** 10.1038/s41598-022-12976-x

**Published:** 2022-05-30

**Authors:** Yeon-Hee Lee, Q-Schick Auh

**Affiliations:** Department of Orofacial Pain and Oral Medicine, Kyung Hee University Dental Hospital, #613 Hoegi-dong, Dongdaemun-gu, Seoul, 02447 Korea

**Keywords:** Health care, Medical research, Risk factors, Signs and symptoms

## Abstract

Chronic pain conditions, including temporomandibular disorders, are closely related to poor sleep quality. This study investigated whether sleep deterioration in patients with painful temporomandibular disorder differed depending on the origin of pain, and also analyzed which clinical disease characteristics and whether psychological distress affected sleep quality. A total of 337 consecutive patients (215 women; mean age, 33.01 ± 13.01 years) with painful temporomandibular disorder (myalgia [n=120], temporomandibular joint arthralgia [n=62], mixed joint–muscle temporomandibular disorder pain [n=155]), who were assessed and classified based on the diagnostic criteria for temporomandibular disorder (DC/TMD), were enrolled. They completed a battery of standardized reports on clinical sign and symptoms, and answered questions on sleep quality, excessive daytime sleepiness, and patients’ psychological status. The mean global Pittsburgh Sleep Quality Index scores were significantly higher in the mixed temporomandibular disorder pain group (6.97 ± 3.38) and myalgia group (6.40 ± 3.22) than in the arthralgia group (5.16 ± 2.94) (*p*=0.001). Poor sleepers were significantly more prevalent in the mixed temporomandibular disorder pain group (76.8%) and myalgia group (71.7%) than in the arthralgia group (54.8%) (*p*=0.006). The presence of psychological distress in the myalgia group (β=1.236, *p*=0.022), global severity index of the Symptom Checklist-90-Revised in the arthralgia group (β=1.668, *p*=0.008), and presence of headache (β=1.631, *p*=0.002) and self-reported sleep problems (β=2.849, *p*<0.001) in the mixed temporomandibular disorder pain group were associated with an increase in the Pittsburgh Sleep Quality Index score. Ultimately, as the source of pain in painful temporomandibular disorder can affect and determine sleep quality and contributing factors, and as the complex interplay between sleep and pain can vary, a comprehensive treatment approach is necessary because good sleep is required by patients.

## Introduction

Humans sleep for a quarter to a third of their lifetime. Although human activity decreases during sleep, sleep is essential for health and pain control. Humans engage in certain productive activities while awake but obtain crucial health benefits during sleep, such as energy conservation, immune function regulation, homeostasis maintenance, memory consolidation, growth and recovery of the body, and psychological relaxation during sleep^1^. Therefore, good sleep quality is a key factor underlying good daytime performance, emotional well-being, physical health, and pain control. Vice versa, poorer sleep quality is predicted by higher pain severity, poorer brain functioning, and greater psychologic distress^2^. Sleep deterioration, pain, and psychological distress are interlinked and inseparable.

Temporomandibular disorder (TMD) is an umbrella term for heterogeneous pain and dysfunction in the temporomandibular joint (TMJ) and masticatory muscle regions. Approximately 39% of the population has at least one sign or symptom of a TMD, and 25% have pain related to a TMD^3^. TMD has a multifactorial etiology. TMD is caused by the accumulation of daily microtraumas in the TMJ area due to parafunctional oral habits, such as bruxism and clenching. However, macrotrauma, psychological distress, and sleep problems also contribute to its development^4^. TMD is best understood within a biopsychosocial framework^5^. Following the publication of the Research Diagnostic Criteria for Temporomandibular Disorders (RDC/TMD) in 1992, the Diagnostic Criteria for TMD (DC/TMD), a revised version that can be used for practical diagnosis in the clinical field, was published in 2014^6^. In TMD diagnosis based on the DC/TMD Axis I, myalgia and arthralgia of the TMJ are the two main sub-diagnoses of TMD. In general, arthralgia is described as a well-defined inflammatory process, and the number of painful joints is related to poor sleep quality^7^. In contrast, chronic myalgia is caused by enigmatic pathophysiological mechanisms considered to be a pain syndrome and which interferes with both the quantity and quality of sleep^8,9^.

The Pittsburgh Sleep Quality Index (PSQI) is an internationally widely used instrument for assessing self-perceived sleep quality and is well validated and reliable. According to previous RDC/TMD- and PSQI-based study, poor sleep quality is found in 60.3% of patients with TMD, and the global PSQI score in painful TMD cases was statistically higher than that in non-painful TMD cases^10^. Yatani et al. reported that up to 90% of patients with TMD diagnosed by RDC/TMD had low sleep quality with high global PSQI^11^. Although the exact mechanism of the complex interaction between TMD pain and poor sleep remains under investigation, some researchers have suggested that painful TMD diagnoses and poor sleep quality are closely related^10^. However, a newly developed DC/TMD-based study on sleep quality in patients with painful TMD and sleep surveys according to the TMD subgroup of DC/TMD has not been conducted so far.

When pain becomes chronic, there is a stronger relationship between sleep deterioration and pain aggravation. Chronic pain has an underlying mechanism that involves a more complex relationship between physical, psychological, and social factors than acute pain^12^. Thus, chronic pain should be understood following a biopsychosocial model. Sleep problems cause musculoskeletal hypersensitivity, and TMD is a representative form of musculoskeletal pain in the orofacial area, which is prone to experiencing chronic pain. Considering the source of TMD pain or TMD subgroups, sleep quality was significantly worse in patients with TMD with myofascial pain than in patients with TMJ intracapsular pain^13^. In addition to poor sleep, psychosocial aspects are related to the experience of pain in cases of painful TMD^14^. Moreover, patients with myogenous TMD had more comorbid disorders including severe headache, fainting, anxiety, depression, and frequent sore throats, and more severe pain than patients with arthrogenous TMD^15^. Unfortunately, few studies have documented whether sleep quality differs according to the chronicity of symptoms, the number of DC/TMD sub-diagnoses, as well as the origin of TMD pain in patients, so novel clinical approaches and scientific evidence addressing these relationships are needed.

Taken together, we hypothesized that sleep quality differs in patients with painful TMD depending on the origin of TMD pain (myalgia, arthralgia, and muscle–joint mixed TMD pain as defined in the DC/TMD), and sleep may be worse when there are multiple origins of pain. Furthermore, we first analyzed the association between poor sleep quality and clinical disease characteristics, excessive daytime sleepiness, and psychological distress in patients with painful TMD, which has not been previously reported. Controlling the risk factors for TMD is the first step in reducing healthcare costs and preventing TMD from becoming chronic. Therefore, it is worth analyzing the factors that contribute to poor sleep quality in patients with painful TMD.

## Materials and methods

### Subjects

A total of 337 consecutive patients (215 women and 122 men; mean age, 33.01 ± 13.01 years) who visited the Department of Orofacial Pain and Oral Medicine of Kyung Hee University Dental Hospital (Seoul, South Korea) over a 6-month period from October 2020 and March 2021 for the management of painful TMD were included in this study. All patients were examined by two TMD specialists with more than 7 years of experience and were carefully examined and diagnosed using the DC/TMD examination. Patients also completed comprehensive questionnaires, including the DC/TMD symptom questionnaire, DC/TMD demographics, oral behavior checklist, and TMD pain screener.

Exclusion criteria were as follows: (1) patients who were under 18 years of age, (2) patients who had other systemic muscular disorders (e.g., fibromyalgia, rheumatoid arthritis, inflammatory joint disease), (3) patients who had neurologic impairment or diseases (e.g., stroke, tumor, or epilepsy), (4) patients who were pregnant, (5) patients who had a history of psychiatric disorders, and (6) patients unable to provide informed consent.

The inclusion criteria were diagnosis of painful TMD according to the DC/TMD Axis I classification, and report of pain at the TMJ and/or masticatory muscles for at least 3 months. A total of 382 patients diagnosed with painful TMD were included, and 45 patients were excluded because they lacked medical documentation. Finally, 337 patients with painful TMD were then divided into three groups as follows: pain of muscle origin (myalgia, n=120), pain of joint origin (arthralgia, n=62), and muscle–joint mixed TMD pain (mixed TMD pain, n=155).

All participants provided written consent for the study, which was approved by the Ethical Committee of Kyung Hee University Dental Hospital (KHD IRB no. 1804-2). The study was conducted in accordance with the principles of the Declaration of Helsinki.

### Data Collection

Experienced orofacial pain specialists conducted comprehensive clinical and radiographic examinations. TMD pain, sleep quality, psychological profile, and excessive daytime sleepiness were assessed using standard, validated, and reliable self-reported instruments administered at the initial evaluation.

#### Characteristics of pain

The duration of pain derived from the TMJ and/or masticatory muscles is reported in days. Temporomandibular pain was scored by the patients subjectively, ranging from 0 (no pain at all) to 10 (worst pain imaginable) using a visual analog scale (VAS).

#### Contributing factors and comorbidities

We investigated self-reported parafunctional activities using the Oral Behavior Checklist, which includes jaw-related behaviors, such as teeth clenching and bruxism. The presence of headache was evaluated using the dichotomous question, “Do you have any headache in the temporal region and headache affected by jaw movement, function, or parafunction?” The presence of self-assessed tinnitus, psychological distress, and sleep problems was also reported with a binary answer. That is, each variable was recorded as a binary answer (yes/no) in all patients, as described in our previous study^16^.

#### Sleep quality evaluation using PSQI

Using the 19-item PSQI, a well-validated self-report questionnaire, sleep quality in the past month was assessed. The PSQI has seven components: subjective sleep quality, sleep latency, sleep duration, sleep efficiency, sleep disturbances, use of sleep medication, and daytime dysfunction. Each subscale is weighted equally, scored from 0 (good sleep/no problems) to 3 (poor sleep/severe problems), summing to a global PSQI score (range, 0–21), with higher scores denoting worse sleep quality^17^. The PSQI was used to measure sleep quality. According to the recommended cutoff point of the PSQI (>5), the patients in this study were categorized as good sleepers (score ≤5) and poor sleepers (score ≥6).

#### Assessment of psychological distress using the symptom checklist-90-revision

The Symptom Checklist-90-Revision (SCL-90R), a validated brief self-report psychometric inventory, has been used to evaluate psychological profiles^18^. It contains 90 items, and patients rated each item on a 5-point scale (not at all, 0; extremely, 4) for how much each problem had distressed or bothered them during the past 7 days. It yielded nine subscale scores, including somatization, obsessive-compulsive, interpersonal sensitivity, anxiety, depression, hostility, phobic anxiety, paranoid ideation, and psychoticism, along with three global indices of distress (Global Severity Index, Positive Symptom Distress Index, and Positive Symptom Total). Higher T-scores of the nine subscales and three global indices on the SCL-90R indicate a greater propensity to experience psychological distress.

#### Excessive daytime sleepiness measured using the epworth sleepiness scale

The Epworth sleepiness scale (ESS) is a validated clinical tool for evaluating excessive daytime sleepiness (EDS)^19^. The ESS is an eight-item, self-administered questionnaire designed to measure the subject’s propensity to fall asleep in a variety of situations. The participant was instructed to answer how likely it is that they would fall asleep in different situations by giving a score on a 4-point scale (0–3). Thus, the total score (the sum of scores of the eight items) of the ESS ranges from to 0–24. Higher scores represent a greater possibility that the individual will fall asleep during the daytime. Unlike other scales that measure sleepiness at a single time point, the ESS is designed to evaluate the general level of sleepiness. The ESS total scores were dichotomized into scores ≤10 and >10; the latter was considered to represent clinically significant EDS. We used a score >10 on the ESS to measure excessive sleepiness.

### Statistical analysis

The data were analyzed using SPSS Statistics for Windows, Version 26.0, (IBM Corp., Armonk, NY, USA). Continuous variables are presented as means and standard deviations (SD), and categorical variables are presented as frequencies and percentages. The inter-rater reliability between the two experts in the diagnosis of painful TMD was assessed using Cohen’s kappa coefficient and was 0.91 for myalgia, 0.94 for arthralgia, and 0.92 for mixed TMD pain groups. If the same diagnosis was not given by the two experts, the patient was finally assigned to one TMD group following in-depth discussion. Differences between the three painful TMD groups were examined using the chi-squared test for categorical variables and t-test and one-way analysis of variance (ANOVA) with Tukey’s post-hoc test for numeric variables. The chi-squared test with Bonferroni adjusted post hoc analysis was used to determine the equality of proportions. Spearman’s correlation coefficients (r) for global PSQI scores with demographics, TMD pain severity, and psychological profiles were calculated, ranging from −1 to +1, with −1 indicating a perfectly linear negative correlation and +1 indicating a perfectly linear positive correlation. Subsequently, assuming that the factors showing a significant correlation with the global PSQI score are predictors, multiple linear regression analysis with a stepwise method was conducted to explore significant predictors for increasing PSQI scores in patients with painful TMD. The estimated β was calculated using multiple linear regression analyses after adjusting for age. For all analyses, a two-tailed p-value of less than 0.05 was considered statistically significant.

### Institutional review board statement

The research protocol was reviewed in compliance with the Helsinki Declaration and approved by the Institutional Review Board of the Kyung Hee University Dental Hospital (KHD IRB no. 1804-2).

### Informed consent

Informed consent was obtained from all subjects involved in the study.

## Results

### General description

Table 1 presents the distribution of demographics, clinical characteristics, and contributing factors for the three TMD groups. A total 337 patients (215 women [63.8%]; mean age, 33.01 ± 13.01 years) were assigned to the TMD group. The composition of patients in our study period with painful TMD according to the DC/TMD Axis I enrolled during the study period included 35.6% with myalgia, 18.4% with arthralgia, and 46.0% with mixed TMD pain. In other words, TMD pain of both muscular and articular origins was the most prevalent. The ratio of men to women was 1:1.76, and the proportion of women with myalgia (60.0%) or mixed TMD pain (80.0%) was significantly higher than the proportion with arthralgia (30.6%) (p<0.001). TMD is thought to be sexual dimorphic and predominantly afflict women^20^. The age distribution was not significantly different between the three groups.

**Table 1:** Demographic characteristics of TMD patients.

Parameter	Myalgia (n=120)	Arthralgia (n=62)	Mixed TMD pain (n=155)		
	n (%) or Mean ± SD	n (%) or Mean ± SD	n (%) or Mean ± SD	Post-hoc	*p*-value
*Demographics*					
Age (years)	33.80 ± 13.36	31.93 ± 13.57	32.81 ± 12.55		0.636
Sex (female %)	72 (60.0)	19 (30.6)	124 (80.0)	1>2, 2<3	<0.001***
*Clinical characteristics*					
VAS	4.49 ± 2.77	4.97 ± 2.54	5.71 ± 1.93	1<3, 2<3	<0.001***
Mouth opening limitation	8 (6.7)	10 (16.1)	20 (12.9)		0.115
Symptom duration (days)	524.35 ± 2010.22	272.94 ± 469.26	620.59 ± 1171.99	1>2, 2<3	0.044*
*Contributing factors*					
Bruxism	35 (29.2)	21 (33.9)	54 (34.8)		0.594
Clenching	49 (40.8)	23 (37.1)	74 (47.7)		0.284
Macrotrauma history	6 (5.0)	4 (6.5)	12 (7.7)		0.659
Tinnitus	34 (28.3)	9 (14.5)	48 (31.0)	2<1, 2<3	0.044*
Headache	45 (37.5)	17 (27.4)	99 (63.9)	1<3, 2<3	<0.001***
Psychological distress	66 (55.0)	30 (48.4)	91 (58.7)		0.381
Self-reported sleep problem	42 (35.0)	19 (30.6)	57 (36.8)		0.694

The results were obtained from χ2 test with Bonferroni adjusted post hoc analysis and the mean difference between groups was obtained by ANOVA) with Tukey’s post-hoc test. *p*-Value significance was set at <0.05. *: *p*-value <0.05, **: *p*-value <0.01, ***: *p*-value <0.001. TMD: temporomandibular disorder, VAS: visual analogue scale, SD: standard deviation, 1 in Post-hoc test: the mean value of myalgia group, 2 in Post-hoc test: the mean value of arthralgia group, 3 in Post-hoc test: the mean value of mixed TMD pain group.

The VAS score was significantly higher in patients with mixed TMD pain (5.71 ± 1.93) than in those with arthralgia (4.97 ± 2.54) and myalgia (4.49 ± 2.77) (*p*<0.001). There was no significant difference in the frequency of mouth opening limitation between groups. The mean symptom duration was significantly longer in the myalgia (524.35 ± 2010.22 days) and mixed TMD pain groups (620.59 ± 1171.99 days) than in the arthralgia group (272.94 ± 469.26 days) (*p*=0.044). Considering the contributing factors, the prevalence of tinnitus and headache were significantly higher in the myalgia and mixed TMD pain groups than in the arthralgia group (*p*<0.05). The prevalence of bruxism, clenching, macrotrauma history, and psychological distress did not differ significantly between the TMD groups. There was no significant difference in the presence of self-reported sleep problems between the myalgia (35.0%), arthralgia (30.6%), and mixed TMD groups (36.8%) (*p*=0.694). Psychological distress was observed in 55.4% of patients with painful TMD and was the most frequently observed contributing factor in all three groups, whereas a history of macrotrauma was only present in 6.5% and the least observed factor in all three groups.

### PSQI in patients with painful TMD

The distribution of PSQI global scores for patients with painful TMD and the proportion of poor sleepers is shown in Table 2.

**Table 2:** Comparison of Pittsburgh Sleep Quality Index (PSQI) between TMD groups.

Parameter	Myalgia (n=120)	Arthralgia (n=62)	Mixed TMD pain (n=155)		
	n (%) or Mean ± SD	n (%) or Mean ± SD	n (%) or Mean ± SD	Post-hoc	*p*-value
*PSQI*					
Component 1: Subjective sleep quality (0–3)	1.28 ± 1.16	1.19 ± 1.40	1.52 ± 1.24		0.108
Component 2: Sleep latency (0–3)	0.83 ± 0.75	0.77 ± 0.76	0.97 ± 0.71		0.118
Component 3: Sleep duration (0–3)	0.92 ± 1.03	0.55 ± 0.72	0.88 ± 0.97	1>2, 3>2	0.035*
Component 4: Sleep efficiency (0–3)	0.57 ± 0.98	0.18 ± 0.46	0.46 ± 0.89	1>2, 3>2	0.016*
Component 5: Sleep disturbances (0–3)	1.22 ± 0.61	1.11 ± 0.66	1.35 ± 0.62	3>2	0.023*
Component 6: Use of sleep medication (0–3)	0.14 ± 0.54	0.03 ± 0.25	0.19 ± 0.59		0.147
Component 7: Daytime dysfunction (0–3)	1.44 ± 0.91	1.32 ± 0.92	1.59 ± 0.86		0.100
PSQI global score (0–21)	6.40 ± 3.22	5.16 ± 2.94	6.97 ± 3.38	1>2, 3>2	0.001**
Poor sleeper (PSQI global score ≥ 5)	86 (71.7)	34 (54.8)	119 (76.8)	1>2, 3>2	0.006**

The results were obtained from χ2 test with Bonferroni adjusted post hoc analysis and the mean difference between groups was obtained by ANOVA) with Tukey’s post-hoc test. *p*-Value significance was set at <0.05. *: *p*-value <0.05, **: *p*-value <0.01. TMD: temporomandibular disorder, VAS: visual analogue scale, SD: standard deviation, 1 in Post-hoc test: the mean value of myalgia group, 2 in Post-hoc test: the mean value of arthralgia group, 3 in Post-hoc test: the mean value of mixed TMD pain group.

Based on the PSQI, poor sleep quality was more pronounced in patients with painful TMD with pain of both joint and muscle origin than in those with only one pain origin. Overall, the PSQI global score was significantly higher in the myalgia (6.40 ± 3.22), and mixed TMD pain (6.97 ± 3.38) groups than in the arthralgia group (5.16 ± 2.94), and the frequency of poor sleepers was significantly higher in the myalgia and mixed TMD groups than in the arthralgia group (all *p*<0.05).

A significant proportion (70.9%) of patients with painful TMD were poor sleepers, and the proportion of poor sleepers was significantly higher among patients with myalgia (71.7%) and mixed TMD pain (76.8%) than among patients with arthralgia (54.8%). The factors affecting sleep quality differed between the three TMD groups. Sleep disturbance occurred more frequently in patients with myalgia than in those with arthralgia, and sleep deterioration was more severe in patients with both diagnoses.

### Psychological distress in patients with painful TMD

Table 3 shows the psychological profiles of patients with painful TMD. Of the nine subdimensions of the SCL-90R, there were significant differences in the mean values of all items except for hostility. Interestingly, the T-scores for somatization, obsessive-compulsive, interpersonal sensitivity, and paranoid ideation were not significantly different between the myalgia and arthralgia groups but were significantly higher in patients with mixed TMD pain than in those with only myalgia or arthralgia. In addition, the mean T-scores for depression, anxiety, phobic ideation, and psychoticism were significantly higher in the mixed TMD pain group than in the myalgia group (all *p*<0.05).

**Table 3:** Comparison of psychological profile with SCL-90R and excessive sleepiness scale between TMD groups.

Parameter	Myalgia (n=120)	Arthralgia (n=62)	Mixed TMD pain (n=155)		
	n (%) or Mean ± SD	n (%) or Mean ± SD	n (%) or Mean ± SD	Post-hoc	*p*-value
*SCL-90R*					
Somatization	45.75 ± 7.39	45.50 ± 8.67	48.59 ± 9.59	1<3, 2<3	0.009**
Obsessive-compulsive	43.65 ± 9.60	42.69 ± 10.04	46.54 ± 10.83	1<3, 2<3	0.015*
Interpersonal sensitivity	43.67 ± 9.46	43.71 ± 10.17	46.75 ± 10.81	1<3, 2<3	0.024*
Depression	45.99 ± 30.31	44.16 ± 11.08	46.86 ± 11.85	1<3	0.676
Anxiety	43.77 ± 7.09	44.44 ± 9.04	46.53 ± 9.86	1<3	0.029*
Hostility	44.61 ± 7.45	45.56 ± 9.04	50.83 ± 35.94		0.098
Phobic anxiety	44.10 ± 4.62	45.55 ± 8.06	47.00 ± 9.97	1<3	0.013*
Paranoid ideation	43.16 ± 8.85	43.06 ± 8.32	45.90 ± 10.12	1<3, 2<3	0.026*
Psychoticism	43.23 ± 6.66	44.56 ± 9.35	45.46 ± 9.28	1<3	0.098
Global severity index	43.05 ± 7.89	43.40 ± 10.19	46.44 ± 10.96	1<3, 2<3	0.010*
Positive symptom distress	45.72 ± 7.44	45.45 ± 8.30	47.62 ± 9.46		0.104
Positive symptom total	41.79 ± 10.56	41.10 ± 12.13	45.01 ± 11.57	1<3, 2<3	0.020*
*ESS*					
Sitting and reading (0–3)	0.98 ± 0.83	0.95 ± 0.69	1.07 ± 0.74		0.483
Watching TV (0–3)	0.68 ± 0.74	0.58 ± 0.67	0.72 ± 0.67		0.400
Sitting, inactive in public place (0–3)	0.55 ± 0.61	0.61 ± 0.66	0.75 ± 0.74	1<3	0.042*
As a passenger in a car for an hour without a break (0–3)	1.18 ± 0.90	1.08 ± 0.84	1.41 ± 0.90	1<3, 2<3	0.017*
Lying down to rest in the afternoon when circumstances permit (0–3)	1.33 ± 0.85	1.44 ± 0.88	1.69 ± 0.92	1<3	0.002**
Sitting and talking to someone (0–3)	0.32 ± 0.59	0.18 ± 0.39	0.39 ± 0.64		0.058
Sitting quietly after a lunch without alcohol (0–3)	1.14 ± 0.87	1.26 ± 0.79	1.48 ± 0.85	2<3	0.004**
In a car, while stopped for a few minutes for traffic (0–3)	0.38 ± 0.55	0.45 ± 0.69	0.52± 0.70		0.243
ESS total score (0–24)	6.56 ± 3.56	6.55 ± 3.50	8.04 ± 3.82	1<3, 2<3	0.001**
ESS total score ≥ 10 (Excessive daytime sleepiness)	23 (19.2)	12 (19.4)	46 (29.7)		0.082

The results were obtained from χ2 test with Bonferroni adjusted post hoc analysis and the mean difference between groups was obtained by ANOVA with Tukey’s post-hoc test. *p*-Value significance was set at <0.05. *: *p*-value <0.05, **: *p*-value <0.01. TMD: temporomandibular disorder, VAS: visual analogue scale, SD: standard deviation, ESS: Epworth sleepiness scale, 1 in Post-hoc test: the mean value of myalgia group, 2 in Post-hoc test: the mean value of arthralgia group, 3 in Post-hoc test: the mean value of mixed TMD pain group.

### EDS in patients with painful TMD

The total ESS score was significantly higher in the mixed TMD pain group (8.04 ± 3.82) than in the myalgia (6.56 ± 3.56) and arthralgia groups (6.55 ± 3.50) (*p*=0.01), whereas the proportion of patients experiencing EDS (ESS >10) did not differ significantly between the three groups (myalgia: 19.2%, arthralgia: 19.4%, mixed TMD pain: 29.7%).

The daytime sleepiness score was higher among patients with mixed TMD pain than in those with myalgia under two conditions: sitting inactive in a public place and lying down to rest in the afternoon when circumstances permit. In the condition of sitting quietly after lunch without alcohol, the degree of daytime sleepiness in the mixed TMD pain group was higher than that in the arthralgia group. In the case of being a passenger in a car for an hour without a break, daytime sleepiness was higher in the mixed TMD group than in the myalgia and arthralgia groups. However, there was no significant difference between groups in the degree of daytime sleepiness in the following four conditions: sitting and reading, watching TV, sitting and talking to someone, and stopping for a few minutes in traffic in a car.

### Correlations between PSQI global scores and other factors

**Figure 1:**
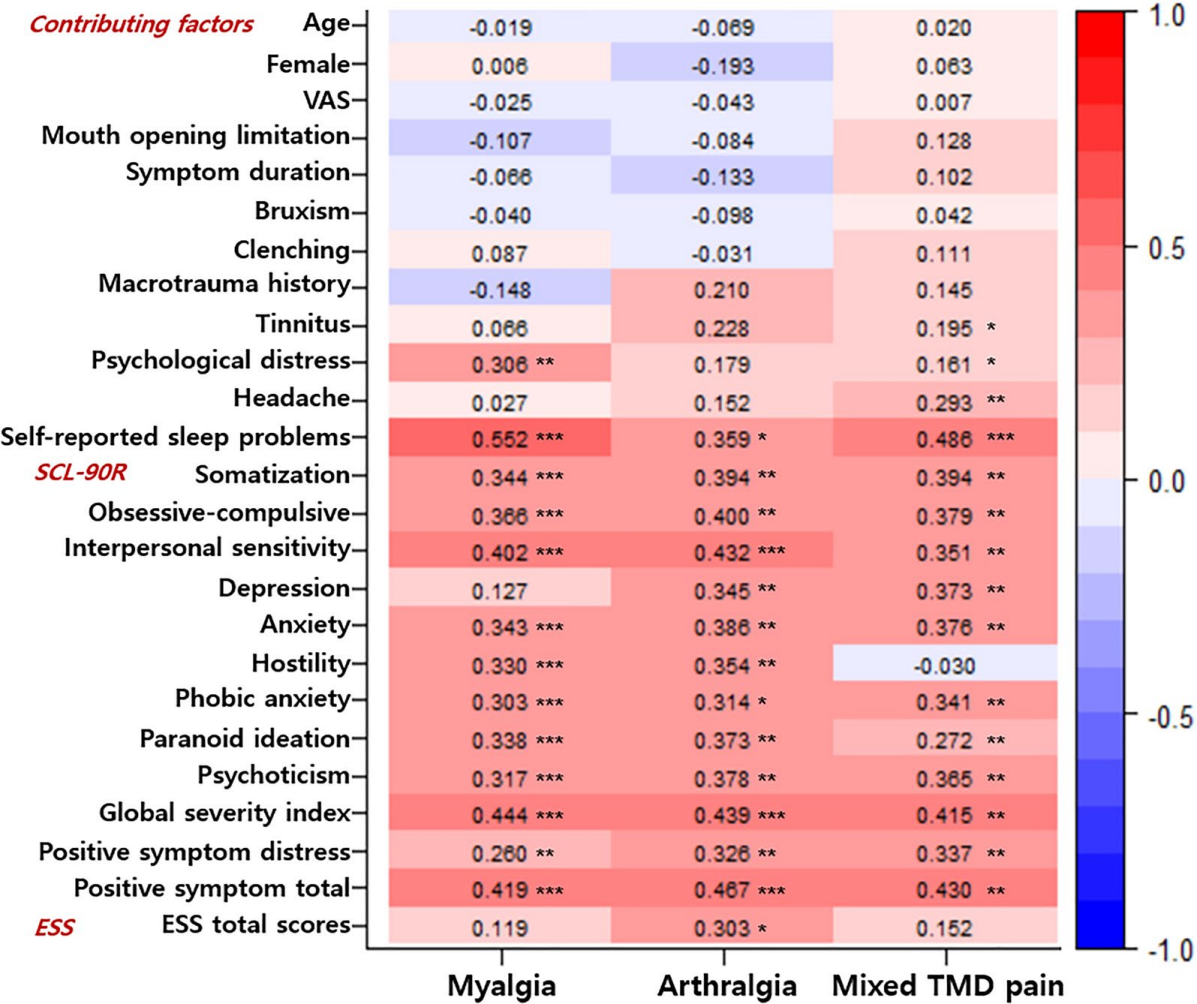
Correlations of PSQI global scores with clinical characteristics and contributing factors for TMD. P-value was considered as significant when p-value < 0.05 (*: *p* < 0.05, **: *p* < 0.01, ***: *p* < 0.001).

Figure 1 shows the results of Spearman’s correlation analysis between the PSQI global scores and all other variables examined in this study. The specific related factors and their correlation strengths were different for each group. In the myalgia group, psychological distress (r=0.306, *p*<0.001) strongly correlated with PSQI global score. In the mixed TMD pain group, tinnitus (r=0.195, *p*=0.015) and headache (r=0.293, *p*=0.001) significantly correlated with PSQI global score. Self-reported sleep problems investigated using dichotomous questions positively correlated with the PSQI global score in all three groups (myalgia: r=0.552, *p*<0.001; arthralgia: 0.359, *p*=0.004; mixed TMD pain: r=0.486, *p*<0.001).

The correlation between the PSQI global score and psychological profiles was remarkable. The psychological profiles of the three groups significantly correlated with sleep quality. Regarding the nine subscales of the SCL-90R, all nine subscales correlated with an increase in PSQI global scores, and all subscales excluding hostility significantly positively correlated with PSQI global scores in the mixed TMD pain group. Total ESS scores significantly positively correlated with PSQI global scores only in the arthralgia group (r=0.303, *p*=0.017).

In all three groups, demographics such as age and sex, VAS, and mouth opening limitation did not show any significant relationship with PSQI global scores.

### Predicting the factors influencing sleep quality

**Figure 2:**
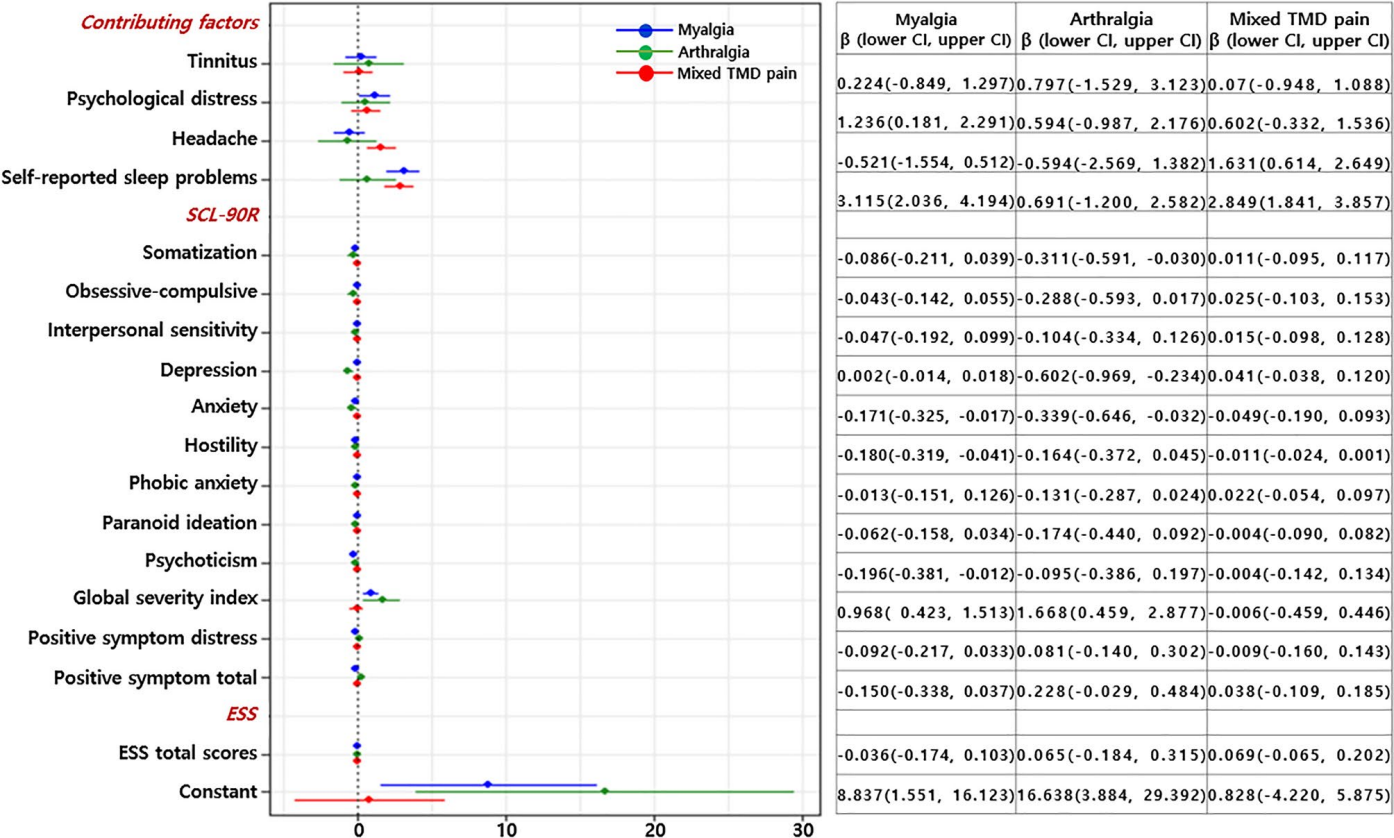
Linear regression analysis when assuming PSQI global scores as an independent variable. Myalgia group: R=0.689, R-squared=0.475, and adjusted R-squared=0.387; Arthralgia group: R=0.675, R-squared=0.455, and adjusted R-squared=0.245; Mixed TMD pain group: R=0.640, R-squared=0.410, and adjusted R-squared=0.337.

Following the Spearman’s correlation analysis, multiple linear regression analysis was performed on 17 predictor variables that significantly correlated with the PSQI global score (Fig. 2). Regarding contributing factors, increased PSQI global scores were predicted by psychological distress (β=1.236, *p*=0.022) and self-reported sleep problems (β=3.115, *p*<0.001) in the myalgia group, and headache (β=1.631, *p*=0.002) and self-reported sleep problems (β=2.849, *p*<0.001) in the mixed TMD pain group. The existence of self-reported sleep problems expressed as a dichotomy (yes or no) was strongly associated with the PSQI global score. In arthralgia, the global severity index of the SCL-90R was a predictor of the PSQI global score only in the arthralgia group (β=1.668, *p*=0.008).

## Discussion

Clinical characteristics differed according to TMD diagnosis based on DC/TMD, and their effect on sleep quality also differed according to the origin of TMD pain. TMD is an umbrella term, and the signs and symptoms of patients with TMD are diverse. As mentioned above, painful TMD is not only observed in patients with myalgia, arthralgia, and mixed TMD pain, but also in patients with aggravated psychological pain as determined by DC/TMD Axis II^21^. Poor sleepers accounted for 70.9% of patients with painful TMD, and the proportion of poor sleepers was significantly higher in the myalgia (71.7%) and mixed TMD pain groups (76.8%) than in the arthralgia group (54.8%). The proportion of poor sleepers was previously reported to be 60.3% among patients with TMD, regardless of the presence of pain^10^.

The PSQI global score was also significantly higher in the myalgia (6.40 ± 3.22) and mixed TMD pain (6.97 ± 3.38) groups than in the arthralgia group (5.16 ± 2.94). The PSQI mean score of patients with painful TMD reported by Rener-Sitar et al. was 7.5, somewhat higher than our result^10^, which may be due to differences in the subjects’ ethnicity, study area, and study methods. In addition, the mixed TMD pain group had the highest VAS score and highest PSQI global score. According to Zamani et al., the sleep quality measured using PSQI deteriorates as the symptom severity of TMD increases^22^. In a recent study, compared to TMD-free controls, painful TMD caused a 3.2 times increase in the frequency of poor sleep as measured using PSQI, which was higher than the 1.91 times in intra-articular TMDs^23^. We hypothesized that sleep quality might differ with painful TMD sub-diagnosis according to the DC/TMD, and our results suggest that sleep and related clinical factors are dependent on the source of TMD pain and symptom severity.

An in-depth discussion on the reason for the difference in sleep quality according to the origin of TMD pain is needed. Among TMD sub-diagnoses, arthralgia is accompanied by a well-defined inflammatory process mediated by inflammatory cytokines, whereas chronic myalgia presents with an enigmatic pathophysiological mechanism^24–26^. In contrast, muscle pain is poorly localized and has a pressing quality, marked tendency towards referral of pain, and more affective aspect; arthralgia is well-localized and has a stabbing quality, no tendency toward referral of pain, and less affective aspect. In general, psychosocial factors, such as depression and tension personality, are more frequently observed in patients with myogenous TMD^27^. Unlike arthralgia, myalgia is often accompanied by changes in psychological state^28,29^. Anxiety and stress-related bruxism activity are positively associated with myogenous TMD pain^27^. In particular, psychosocial forces can shape individual vulnerability to pain-related outcomes over time^30^. Therefore, analgesic and anti-inflammatory drugs are prescribed for arthrogenous pain; however, antidepressants, anticonvulsants, and muscle relaxants are commonly prescribed for myogenous pain^31^. Although some patients with advanced osteoarthritis develop central sensitization, central sensitization and neuropathic features have been reported in patients with muscle pain^32^.

The exact etiology of the central mechanism of muscle pain and its relationship with psychological distress remain unclear. Starting with a peripheral mechanism of myalgia excitability, the outlasting sensitization of peripheral nociceptors may result in persistent hypersensitivity of peripheral nociceptors, which are involved in excitation and/or dysfunction of central neurons^33^. As a next step, increased muscle tension can be a local trauma, physiological dysfunction, and lead to a defensive response to psychological burden^34^. A central mechanism in the development of muscle pain due to prolonged overactivity is primarily concerned with the muscles that tense when experiencing psychological discomfort, anxiety, or stress^35^. Specifically, the masseter muscle, temporal muscle, sternocleidomastoid muscle, and trapezius muscle are typical examples mentioned when discussing the central mechanism. These muscles tend to respond to changes in mental state that can cause muscle pain associated with mental disorders or chronic stress^36^. Poor sleep is most likely a comorbidity or a contributing factor that continues their vicious cycle^9^. The disparity between the ability to control muscle pain, good sleep, and mental health can affect the chronicity of symptoms.

As hypothesized, sleep was more disturbed in the mixed TMD pain group with both joint- and muscle-derived pain than in those with pain from a single origin. This suggests that when symptoms are complex with multiple origins of pain, sleep and psychosocial aspects are more vulnerable. According to Trivedi et al., as the number of physical symptoms a patient has increases, the morbidity of depression also increases^37^. When there are multiple causes of TMD pain, the psychological aspects can be more complex. Patients with multiple TMD diagnoses have higher rates of depression and somatization^38^. Manfredini et al. reported a tendency to have higher SCL-90-R scores in patients with myofascial pain combined with TMJ pain than in those with TMJ pain alone^39^. However, Reissmann et al. reported that the location of pain in TMD patients was not a major factor when predicting psychosocial profiles and current pain severity^40^. The discrepancy between our results and those of Reissmann et al. might be explained by the fact that they did not consider symptom duration and pain chronicity as the study’s main variables.

In the present study, the mixed TMD pain group had the longest symptom duration and the highest PSQI global score among the three painful TMD groups. The chronicity of TMD pain can be closely linked to poor sleep quality in patients with painful TMD. In general, the prevalence of chronic pain ranges from 10 to 40%^41^, which is similar to the prevalence of sleep disorders (10–36%)^42^. Sleep disorders have been found to affect 88% of patients with chronic pain^43^. Conversely, more than 40% of patients with sleep disorders report chronic pain^44^. In addition, chronic pain and sleep problems commonly have psychological comorbidities, especially depression^45^. As acute pain progresses to the chronic phase, the central pain control system and psychosocial factors can also be more impaired^30^. Regarding changes in the brain, dysregulation of neurotransmitters in the brain is linked to both chronic pain and depression, and this linkage makes pain patterns more complex and exacerbates pain^46^. Chronic pain is also related to anatomical alterations in brain regions involved in cognitive and emotional modulation of pain^47^. There may be a brain circuit that responds differently to noxious stimuli in patients with chronic painful TMD compared to those with acute TMD or non-painful TMD. These complex interactions may explain why patients with TMD pain of mixed origin develop psychological distress and are at increased risk of sleep problems and central amplification of pain. However, studies on the etiopathology and underlying mechanisms of mixed TMD pain based on DC/TMD are extremely limited, and more detailed studies are needed.

Female sex is a major risk factor for both chronic pain and poor sleep quality. In the myalgia and mixed TMD pain groups, the proportion of women was higher than that in the arthralgia group. Women are more exposed to chronic pain than men and are more prone to co-occurrence of chronic pain with depression and anxiety^12^. In a previous study using the SCL-90R in TMD patients, depression and somatization scales were higher in women than in men^48^. In addition, there is a tendency for increased prevalence of tinnitus in patients with chronic pain^49^. In the present study, the prevalence of tinnitus and headache was highest in the mixed TMD pain group. Chronic pain and tinnitus have several similarities and are frequently associated with hypersensitivity to sensory stimuli. Both are abnormal and variable subjective sensations, often not fully explained by initial peripheral lesions; thus, they are referred to as phantom sensations. Moreover, patients suffering from these two conditions often share the same psychological traits with an increased propensity for anxiety and depression^50,51^. Although research on the exact mechanism is needed, the overlap of pain and psychological distress is particularly pronounced in patients with chronic pain and headache^52^.

Both chronic pain and sleep disturbances share an array of psychological health issues. Although the degree of correlation varied between the painful TMD groups, the T-score of the nine parameters of the SCL-90R positively correlated with the PSQI global score. The relationships between pain, poor sleep phenomena, and psychological distress should be considered “reciprocal” in painful TMD. Disturbed sleep affects pain perception by lowering the pain threshold, and patients with pain have poor sleep in terms of sleep efficiency, sleep latency, and awakenings after sleep onset^9^. Furthermore, as pain becomes chronic, central neural plasticity and central sensitization increase, and psychological factors may become vulnerable^53^. Specific psychosocial characteristics place individuals at elevated or reduced risk for the transition from an acute to a persistent pain state^54^. Of course, psychosocial processes exist within an individual as a pre-existing vulnerability factor. Sleep problems considering the chronicity of TMD pain should be further studied in patients with painful TMD.

Our study has several limitations. We did not use investigations such as polysomnography (PSG) or actinography, which are considered the gold standard for sleep evaluation. Especially, PSG presents the results of objectively measuring various aspects of sleep. However, as in many countries around the world, it was not possible to conduct PSG because of the lack of sleep labs, insurance coverage, high cost, and lack of suitability for large-scale studies. PSG has a weakness in that it cannot be performed in a comfortable environment, such as at home, where the patient sleeps every day. Thus, it only reflects the results obtained in the laboratory for just a few days. In addition, there are physical and time limitations for PSG-based research in many patients. Our study was designed as a retrospective study to investigate the conditions under which sleep quality deteriorates in patients with painful TMD; therefore, the causal relationship cannot be clarified. It merely suggests statistically related factors. However, we assessed the sleep of 337 patients with painful TMD using the PSQI, a sophisticated and reliable questionnaire. Although TMD pain was classified according to the DC/TMD criteria, not all subgroups of DC/TMD were analyzed, and the new questionnaire included in DC/TMD AXIS II was not accompanied, so there may be limitations in the interpretation of the results. Subsequent large-scale longitudinal studies and prospective study designs with all subgroups of DC/TMD AXIS I and questionnaires from AXIS II are needed to clarify our findings.

## Conclusions

Successful TMD treatment is possible by considering sleep quality in patients with painful TMD. As sleep problems can be the underlying factors for the onset, development, and prolonging of TMD along with psychosocial profiles, these factors should be examined along with clinical symptoms in patients with painful TMD. In this study, sleep quality differed according to the chronicity of symptoms, number of DC/TMD sub-diagnoses, and origin of TMD pain in patients. In particular, chronic symptom duration, multiple origins of pain, myogenous TMD pain, and psychological distress were associated with worsening sleep quality. Therefore, these sleep and pain-related phenomena must be considered when diagnosing and treating patients with painful TMDs. Global efforts are needed to present treatment guidelines regarding sleep for each subgroup according to the DC/TMD in addition to efforts to increase the accuracy of subgroup diagnosis.

## Data Availability

Since these are patient data, if there is a request for data disclosure, the KHU-IRB will discuss the request before disclosure.
